# Implementation Strategies to Promote Short-Course Radiation for Bone Metastases

**DOI:** 10.1001/jamanetworkopen.2024.11717

**Published:** 2024-05-24

**Authors:** Erin F. Gillespie, Patricia Mae G. Santos, Michael Curry, Talya Salz, Nirjhar Chakraborty, Michael Caron, Hannah E. Fuchs, Nahomy Ledesma Vicioso, Noah Mathis, Rahul Kumar, Connor O’Brien, Shivani Patel, David M. Guttmann, Jamie S. Ostroff, Andrew L. Salner, Joseph E. Panoff, Alyson F. McIntosh, David G. Pfister, Max Vaynrub, Jonathan T. Yang, Allison Lipitz-Snyderman

**Affiliations:** 1 Department of Radiation Oncology, Memorial Sloan Kettering Cancer Center, New York, New York; 2 Department of Radiation Oncology, University of Washington School of Medicine, Fred Hutchinson Cancer Center, Seattle; 3 Department of Epidemiology and Biostatistics, Memorial Sloan Kettering Cancer Center, New York, New York; 4 Department of Strategic Partnerships, Memorial Sloan Kettering Cancer Center, New York, New York; 5 Department of Radiation Oncology, Miami Cancer Institute, Baptist Health South Florida, Miami; 6 Department of Radiation Oncology, Hartford HealthCare Cancer Institute, Hartford, Connecticut; 7 Department of Radiation Oncology, Lehigh Valley Cancer Institute, Allentown, Pennsylvania; 8 Department of Psychiatry and Behavioral Sciences, Memorial Sloan Kettering Cancer Center, New York, New York; 9 Department of Medicine, Memorial Sloan Kettering Cancer Center, New York, New York; 10 Department of Surgery, Orthopaedic Service, Memorial Sloan Kettering Cancer Center, New York, New York; 11 Department of Radiation Oncology, NYU School of Medicine, New York, New York

## Abstract

**Importance:**

For patients with nonspine bone metastases, short-course radiotherapy (RT) can reduce patient burden without sacrificing clinical benefit. However, there is great variation in uptake of short-course RT across practice settings.

**Objective:**

To evaluate whether a set of 3 implementation strategies facilitates increased adoption of a consensus recommendation to treat nonspine bone metastases with short-course RT (ie, ≤5 fractions).

**Design, Setting, and Participants:**

This prospective, stepped-wedge, cluster randomized quality improvement study was conducted at 3 community-based cancer centers within an existing academic-community partnership. Rollout was initiated in 3-month increments between October 2021 and May 2022. Participants included treating physicians and patients receiving RT for nonspine bone metastases. Data analysis was performed from October 2022 to May 2023.

**Exposures:**

Three implementation strategies—(1) dissemination of published consensus guidelines, (2) personalized audit-and-feedback reports, and (3) an email-based electronic consultation platform (eConsult)—were rolled out to physicians.

**Main Outcomes and Measures:**

The primary outcome was adherence to the consensus recommendation of short-course RT for nonspine bone metastases. Mixed-effects logistic regression at the bone metastasis level was used to model associations between the exposure of physicians to the set of strategies (preimplementation vs postimplementation) and short-course RT, while accounting for patient and physician characteristics and calendar time, with a random effect for physician. Physician surveys were administered before implementation and after implementation to assess feasibility, acceptability, and appropriateness of each strategy.

**Results:**

Forty-five physicians treated 714 patients (median [IQR] age at treatment start, 67 [59-75] years; 343 women [48%]) with 838 unique nonspine bone metastases during the study period. Implementing the set of strategies was not associated with use of short-course RT (odds ratio, 0.78; 95% CI, 0.45-1.34; *P* = .40), with unadjusted adherence rates of 53% (444 lesions) preimplementation vs 56% (469 lesions) postimplementation; however, the adjusted odds of adherence increased with calendar time (odds ratio, 1.68; 95% CI, 1.20-2.36; *P* = .003). All 3 implementation strategies were perceived as being feasible, acceptable, and appropriate; only the perception of audit-and-feedback appropriateness changed before vs after implementation (19 of 29 physicians [66%] vs 27 of 30 physicians [90%]; *P* = .03, Fisher exact test), with 20 physicians (67%) preferring reports quarterly.

**Conclusions and Relevance:**

In this quality improvement study, a multicomponent set of implementation strategies was not associated with increased use of short-course RT within an academic-community partnership. However, practice improved with time, perhaps owing to secular trends or physician awareness of the study. Audit-and-feedback was more appropriate than anticipated. Findings support the need to investigate optimal approaches for promoting evidence-based radiation practice across settings.

## Key Points

**Question:**

Is a multicomponent set of implementation strategies (guidelines, electronic consultations [eConsults], and audit-and-feedback reports) associated with improved uptake of short-course radiation within an academic-community partnership?

**Findings:**

In this quality improvement study conducted across 3 community-based cancer centers, a multicomponent set of strategies was not associated with increased use of short-course radiation for nonspine bone metastases. However, short-course radiation practice significantly improved with time, perhaps owing to secular trends or physician awareness of the study.

**Meaning:**

These findings demonstrate a need to examine approaches for encouraging uptake of short-course radiation and other evidence-based practices to improve care among patients with nonspine bone metastases.

## Teaser

This quality improvement study examines whether 3 implementation strategies (guidelines, electronic consultations, and audit-and-feedback reports) are associated with increased adoption of a consensus recommendation to treat patients with nonspine bone metastases with short-course radiotherapy.

## Introduction

Radiation therapy (RT) for nonspine bone metastases is rapidly evolving. Although management of painful nonspine bone metastases historically included use of longer courses (ie, ≥10 radiation treatments or fractions), multiple randomized trials have demonstrated equivalent pain relief with a single treatment,^1^ whereas regimens consisting of up to 5 fractions have also proven efficacious with perhaps lower rates of retreatment.^2,3,4,5^ Meanwhile, emerging data support the safety and efficacy of stereotactic body radiotherapy, which also facilitates shorter treatments.^6,7,8,9,10^ Importantly, evidence suggests that as few as 2 additional radiation treatments can worsen financial toxicity.^11^ Collectively, these findings have led to national consensus guidelines recommending against longer courses of RT.^12,13^

Despite these efforts, up to 30% of patients with cancer in the US received more than 10 fractions for bone metastases in 2017.^14^ Variations in uptake of short-course RT has been attributed to misaligned financial incentives and higher retreatment rates with single-fraction RT.^14,15,16,17^ Meanwhile, implementation strategies, including the dissemination of local or internal guidelines and audit and feedback, have been shown to be effective at improving uptake of some evidence-based practices^18,19,20,21^; however, limited data exist regarding their utility in promoting short-course RT, and none have been evaluated prospectively. Therefore, there is a need to test implementation strategies that may help facilitate high-quality radiation care, particularly among the most vulnerable populations.^22,23^

Building on consensus recommendations developed and published by our study team to help standardize treatment of patients with nonspine bone metastases (including preferred use of 5 fractions or fewer),^24^ we designed a set of implementation strategies to align the clinical practice of radiation oncologists treating nonspine bone metastases across an existing academic-community partnership, known as the Memorial Sloan Kettering (MSK) Cancer Alliance. In the Alliance Group Initiative for Bone Metastases (ALIGNMENT) study, we assessed the feasibility, acceptability, and appropriateness of this set of implementation strategies, as well as the extent to which these strategies could improve uptake of evidence-based practice (ie, short-course RT) for nonspine bone metastases.

## Methods

### Study Design

This stepped-wedge, cluster randomized quality improvement study was conducted between October 2021 and May 2022 to assess the effectiveness of 3 implementation strategies. Rollout was randomized and initiated in 3-month increments across 3 community-based cancer centers within the MSK Cancer Alliance. Effectiveness and outcomes were assessed both before and after implementation, as described later in this article. The ALIGNMENT study followed the Consolidated Standards of Reporting Trials (CONSORT) reporting guidelines, and the current quality improvement study followed the Standards for Reporting Implementation Studies (StaRI) Statement guideline.^25^ The intervention and physician surveys were deemed exempt from human participants review, and analysis of the patient-level data was approved by institutional review boards at all MSK Cancer Alliance sites. Patient consent was waived because the data were deidentified, in accordance with 45 CFR §46.

### Implementation Strategies

The study intervention was a set of implementation strategies designed to promote the use of short-course radiation. With our prior population-based research^26^ suggesting greater variation in adoption of short-course radiation at the physician and institution levels than the patient level, the Consolidated Framework for Implementation Research (CFIR) was used by physicians on the study team (including participating site champions) to systematically consider barriers at each level. Relevant constructs identified for this evidence-based practice included knowledge and/or beliefs, self-efficacy, and individual stage of change, further informing strategy selection targeting the individual physician level. The following 3 physician-level strategies were therefore selected from the Expert Recommendations for Implementing Change^27^ and additional literature review,^18,19,20^ with consideration for local context and preferences provided by site champions: (1) dissemination of professional educational materials (ie, electronic PDF of multidisciplinary consensus guidelines on the management of nonspine bone metastases)^24^; (2) personalized audit-and-feedback reports, which described physician rates of short-course RT use during the baseline period, compared with peers at the same institution and institution-based aggregates from the other sites and MSK; and (3) an email-based consultation platform for community partners to request input from subspecialty experts for difficult cases called eConsults (see eAppendix 1 in Supplement 1 for additional information regarding each strategy).

### Study Setting

The trial was conducted within the MSK Cancer Alliance, a partnership between a tertiary academic (ie, MSK) and 3 community-based cancer centers (ie, Hartford Healthcare, Hartford, Connecticut; Lehigh Valley Health Network, Allentown, Pennsylvania; and Miami Cancer Institute, Miami, Florida) that was established in 2014 to increase clinical trial enrollment among patients receiving care in the community. To facilitate testing of novel therapeutics, the partnership developed processes for clinical practice evaluation (including audit and feedback at the institution level) and established research agreements.^28^ Thus, the MSK Cancer Alliance offered a unique, although increasingly common, context within which to study strategies to improve delivery of RT in the community. Although the MSK Cancer Alliance had not historically used specific metrics to evaluate radiation practice for bone metastases, the partnership had recently enhanced efforts to support therapeutic clinical trial enrollment in radiation oncology, as described elsewhere.^29^ Practice-level clusters included all 3 community-based sites, each consisting of at least 1 hospital and affiliated outpatient clinics. Each site appointed a radiation oncologist as site champion.

### Conceptual Frameworks to Evaluate Implementation Strategies

The evaluation framework developed by Proctor et al^30^ was used to measure implementation effectiveness outcomes, with adoption assessed at the physician level and penetrance assessed at the bone metastasis lesion level (detailed later). Physician survey questions based on this framework assessed the acceptability, appropriateness, and feasibility of each implementation strategy as defined later. Separately, the CFIR was also used to develop survey questions assessing context and determinants to inform data collection about participating sites.^31,32^

### Effectiveness Assessment

#### Patients, Data Sources, and Variables

The effectiveness of implementation strategies was assessed using clinical data from all patients who underwent RT for nonspine bone metastases at participating sites during the study period. Clinical data for each patient were abstracted from the electronic health record. Radiation data elements included course identifiers (usually named by the anatomic location of the nonspine bone metastasis treated); RT start date (year), dose, technique (ie, 2-dimensional conventional, 3-dimensional conformal, intensity modulated radiation, or stereotactic body radiotherapy); and number of fractions. Patients with course identifiers specifying spinal location (ie, cervical, thoracic, or lumbar) were excluded. In addition, patient age, sex, race and ethnicity, zip code, performance status (Karnofsky Performance Status or Eastern Cooperative Oncology Group), cancer type, number of metastatic sites (oligometastatic, or ≤5 sites), prior radiation or surgery to the treated area, and treating physician were extracted from the medical records. Data on race and ethnicity were included to ensure equitable implementation.

The primary outcome was adherence to the consensus recommendation of short-course RT (ie, ≤5 fractions) for the treatment of nonspine bone metastases.^24^ Patients with multiple lesions were counted by each separate course of treatment.

### Implementation Assessment

#### Survey Participants and Administration

All radiation oncologists at the 3 institutions treating patients with nonspine bone metastases during the study period were invited via email to complete 2 anonymous implementation-related surveys: one before and one after rollout of the implementation strategies (survey instruments are shown in eAppendix 2 in Supplement 1). Site champions encouraged participation with at least 2 reminder emails. Physicians were compensated with a $50 gift card for their effort completing each survey.

#### Survey Development and Implementation Outcome Measures

Survey questions based on the CFIR were designed to assess contextual determinants influencing implementation outcomes, allowing for the systematic and formal evaluation of barriers or facilitators to implementation. Specific questions were identified using the CFIR–Organizational Resource and Context Assessment matching tool.^33^ Contextual determinants of implementation outcomes were assessed across 4 CFIR domains: (1) the characteristics of the evidence-based practice being implemented into the organization, (2) the inner setting (perceived contexts of a given site), (3) the outer setting (perceived contexts of the environment within which a given site resides), and (4) the individuals involved with implementation of short-course RT.

In addition, survey questions based on the Proctor et al^30^ framework were included to assess implementation outcomes.^34^ For acceptability, physicians were asked the degree to which they welcome a given strategy. For appropriateness, physicians were asked the degree to which they agreed that a given strategy was useful and seems like a good match. Finally, for feasibility, physicians were asked the degree to which they felt that a strategy seems implementable and easy to use. The postimplementation survey also contained questions regarding adoption or uptake of each strategy.

### Statistical Analysis

Data analysis was performed from October 2022 to May 2023. For patient data, mixed fixed-effects logistic regression models were used to assess whether the multicomponent set of implementation strategies was associated with increased use of short-course RT at the lesion level. Fixed effects included calendar time, unique institution, preimplementation vs postimplementation status, and patient-level covariates. Physician-level fixed effects included (1) whether a physician treated at least 75% of their patients in adherence with recommendation of 5 fractions or fewer (binary baseline adherence), (2) high vs low patient volume (defined according to relative distribution across all centers), (3) years of experience, and (4) site champion status. The patient’s treating physician was considered a random effect. Odds ratios (ORs) of adherence and 95% CIs represent the difference between exposure levels of lesions treated by the same physician or a physician with an equal random effect.

We expected to obtain a minimum of 24 patients with a diagnosis of nonspine bone metastases per 3-month period at each of the 3 participating institutions. We assumed that the distribution of patient characteristics would be similar across time periods. For this stepped-wedge randomized quality improvement study, there was 90% power to observe an anticipated increase from a baseline of 50% to 75% with 5 or fewer fractions after implementation.

For physician surveys, summary statistics, including measures of central tendency (ie, mean or median) and variance (ie, SD or IQR), were used to describe differences in response between the presurvey and postsurvey. Preimplementation and postimplementation survey responses were compared at the summary level to confirm stability of CFIR constructs using the Fisher exact test. A 2-sided *P* < .05 was considered statistically significant. All statistical analyses for both the effectiveness and implementation assessments were conducted using R statistical software version 4.2.0 (R Project for Statistical Computing).^35^

## Results

### Effectiveness Assessment

#### Patient and Physician Cohort Characteristics

Summary statistics of patient, lesion, and physician characteristics are shown in Table 1 and eTables 1, 2, and 3 in Supplement 1. Overall, 714 patients with 838 unique nonspine bone metastases were treated by 45 physicians across all sites. The median (IQR) age of patients at treatment start date was 67 (59-75) years, and 343 patients (48%) were women. Among patients with available race and ethnicity data, 8 (1.1%) were Asian, 63 (8.8%) were Black, 175 (25.0%) were Hispanic, 581 (82.0%) were White, and 53 (7.4%) were other (ie, American Indian or Alaska Native, multiracial, and unknown). A median (IQR) of 13 (7-20) patients were treated by each physician, whose median (IQR) experience at the time of the study was 21 (10-35) years. Baseline physician adherence to consensus guidelines for 1 year before randomization was available for two-thirds of physicians (30 physicians); 8 physicians adhered to the guideline recommendation at baseline.

**Table 1.** Cohort Characteristics at the Bone Metastasis Lesion Level

Characteristics^a^	Patients, No. (%)	
	Preimplementation	Postimplementation
No. of bone metastasis lesions	628	210
Sociodemographic characteristics		
Age at treatment start, median (IQR), y	66 (59-74)	67 (60-76)
Sex		
Female	299 (48)	104 (50)
Male	329 (52)	106 (50)
Race		
Asian	8 (1.3)	1 (0.5)
Black	54 (8.7)	21 (9.8)
Hispanic	167 (27)	39 (20)
White	516 (83)	169 (83)
Other^b^	45 (7.2)	17 (8.3)
Clinical characteristics		
Institution		
Hartford Healthcare, Hartford, CT	130 (62)	265 (42)
Lehigh Valley Health Network, Allentown, PA	43 (20)	140 (22)
Miami Cancer Institute, Miami, FL	37 (18)	223 (36)
Eastern Cooperative Oncology Group status		
0	152 (25)	59 (29)
1	261 (42)	89 (43)
≥2	204 (32)	58 (27)
>5 metastatic sites	532 (85)	176 (85)
Symptomatic from bone metastasis	194 (96)	591 (95)
Radioresistant	45 (22)	157 (26)
Treatment characteristics		
RT technique		
Complex	34 (17)	94 (15)
Simple	172 (83)	534 (85)
Total planned RT dose, median (IQR), Gy	27 (20-30)	30 (20-30)
Completed planned RT course	191 (93)	603 (96)
Prior RT to currently treated site	17 (8)	41 (6)
Prior surgery to treated bone metastasis	23 (11)	54 (9)

Abbreviation: RT, radiotherapy.

^a^ Data were unknown for Eastern Cooperative Oncology Group (14 patients [1.9%]), race (9 patients [1.3%]), and lesion level (4 patients [0.5%]).

^b^ Other includes American Indian or Alaska Native, multiracial, and unknown.

#### Effectiveness of Implementation Strategies to Change RT Practice

There was no significant difference in the unadjusted proportion of lesions treated with short-course RT (defined as ≤5 fractions) preimplementation vs postimplementation (444 lesions [53%] vs 469 lesions [56%]). See the Figure for unadjusted quarterly proportions over time. The odds of short-course RT treatment among preimplementation lesions were still not significantly different than those treated postimplementation (OR, 0.78; 95% CI, 0.45-1.34; *P* = .40) (Table 2) after controlling for time in study, patient-level and physician-level covariates, and dependence of lesions with the same treating physician. However, adherence to these recommendations improved over time, with higher odds (OR, 1.68; 95% CI, 1.20-2.36; *P* = .003) of adherence among lesions with an additional year of calendar time-in-study compared with a lesion with 1 less year time in study (eg, 1 vs 2 years since study initiation), given that both lesions were treated by the same physician. Odds of adherence at the bone metastasis lesion-level were lower (OR, 0.19; 95% CI, 0.06-0.59; *P* = .004) among those treated by physicians who were nonadherent at baseline than those of their baseline-adherent counterparts.

**Figure.**
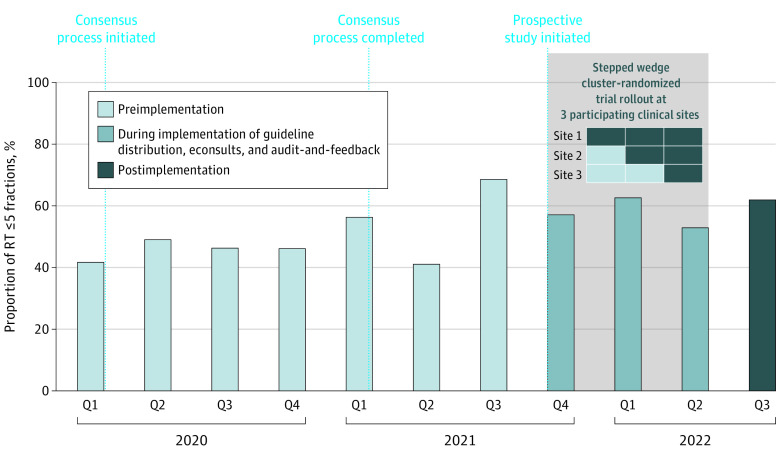
Unadjusted Rates of Short-Course Radiation Therapy (RT) for Nonspine Bone Metastases Bar graph depicts the proportion of radiation courses for nonspine bone metastases consisting of 5 fractions or fewer (ie, short course) by quarter from 2020 to 2022. Unadjusted rates are provided, including during the stepped wedge study rollout in which data are contributed simultaneously (according to clinical site) before and after implementation of the set of strategies. Overall, these unadjusted rates are consistent with the multivariable analysis confirming a general increase in utilization of short-course radiation across participating sites, but not an increase in response to the rollout of implementation strategies. Q indicates quarter.

**Table 2.** Bone Metastasis Lesion-Level Analysis of Effect of ALIGNMENT Strategies on Short-Course Radiotherapy^a^

Characteristic	Univariable regression		Multivariable regression	
	OR (95% CI)	*P* value	OR (95% CI)	*P* value
Preimplementation vs postimplementation (n = 834 lesions)				
Preimplementation	1 [Reference]	NA	1 [Reference]	NA
Postimplementation	1.38 (0.94-2.03)	.10	0.78 (0.45-1.34)	.40
Time in study (n = 834 lesions)	1.51 (1.19-1.92)	<.001	1.68 (1.20-2.36)	.003
Age (n = 833 lesions)	1.01 (1.00-1.02)	.049	1.01 (1.00-1.03)	.06
Sex (n = 833 lesions)				NA
Female	1 [Reference]	NA	NA	NA
Male	1.02 (0.74-1.41)	.90	NA	NA
Race (n = 827 lesions)				
Black	0.68 (0.39-1.18)	.20	0.72 (0.39-1.30)	.30
White	1 [Reference]	NA	1 [Reference]	NA
Other^b^	1.45 (0.82-2.59)	.20	1.89 (0.98-3.63)	.06
Ethnicity (n = 824 lesions)				
Non-Hispanic	1 [Reference]	NA	1 [Reference]	NA
Hispanic	0.98 (0.64-1.52)	>.99	0.71 (0.43-1.17)	.20
Eastern Cooperative Oncology Group status (n = 823 lesions)				
0	1 [Reference]	NA	NA	NA
1	0.93 (0.62-1.38)	.70	NA	NA
≥2	0.86 (0.56-1.33)	.50	NA	NA
Radioresistant (n = 817 lesions)				
Radioresistant	1 [Reference]	NA	1 [Reference]	NA
Radiosensitive	0.86 (0.59-1.26)	.40	0.78 (0.53-1.17)	.20
Physician volume (n = 834 lesions)				
Low volume	1 [Reference]	NA	NA	NA
High volume	1.28 (0.56-2.92)	.60	NA	NA
Physician years of experience (n = 795 lesions)	1.00 (0.97-1.02)	.70	NA	NA
Adherence with recommendation at baseline (n = 834 lesions)				
Adhered at baseline	1 [Reference]	NA	1 [Reference]	NA
Did not adhere at baseline	0.23 (0.08-0.68)	.008	0.19 (0.06-0.59)	.004
No baseline data	0.35 (0.11-1.12)	.08	0.29 (0.08-0.99)	.047
Physician member of study team (n = 834 lesions)	2.71 (0.67-11.0)	.20	3.50 (0.82-14.9)	.09

Abbreviations: NA, not applicable; OR, odds ratio.

^a^ Mixed-effects logistic regression model, with a random effect for physician to assess impact of the implementation strategies on use of short-course radiation (5 or fewer treatments). An OR greater than 1 indicates increased adherence to the recommendation.

^b^ Other includes American Indian or Alaska Native, multiracial, and unknown.

### Implementation Assessment

#### Context and Determinants

Overall, 38 physicians were available to be contacted to complete surveys, of whom 29 (76%) responded preimplementation and 30 (79%) responded postimplementation, with 80% (23 physicians) completing both. Among those surveyed, 16 physicians (53%) reported that their clinical practice was limited to 1 to 3 disease sites. In the past 3 months, 7 physicians (24%) reported treating 10 or more bone metastases and 12 (41%) reported treating 5 to 9 bone metastases. Physician survey responses regarding implementation context and determinants by CFIR domain and subdomain are summarized in Table 3.

**Table 3.** Context and Determinants Assessing Factors in the Clinical Setting That May Be Associated With Implementation and Effectiveness Outcomes

CFIR construct domains and subdomains	Survey question	Response	Physicians, No./total No. (%)^a^		*P* value^b^
			Presurvey	Postsurvey	
Intervention characteristics					
Evidence strength and quality	Please rate the strength of evidence for using ≤5 fractions of RT for nonspine bone metastases.	Moderately strong or strong	24/29 (83)	26/30 (87)	.73
Relative advantage	Using ≤5 fractions of RT to treat nonspine bone metastases appears to have more advantages than disadvantages.	Agree or strongly agree	NA	26/29 (90)	NA
Inner setting					
Networks and communication	Please rate the effectiveness of communication between physicians in your organization and physicians at MSK.	Effective or most effective	12/29 (41)	19/30 (63)	.12
Implementation climate, tension for change	In general, how receptive are staff members in your organization to change in clinical processes?	Receptive or very receptive	28/29 (97)	29/29 (100)	>.99
Readiness for implementation, leadership engagement	Senior leadership in your organization seek ways to improve patient education and increase patient participation in treatment.	Agree or completely agree	NA	22/30 (73)	NA
Outer setting, patient needs and resources	Using ≤5 fractions to treat nonspine bone metastases takes into consideration the needs and preferences of patients.	Agree or strongly agree	NA	28/29 (97)	NA
Individuals, knowledge and beliefs about the intervention	How confident do you feel in your practice treating bone metastases?	Very confident	24/29 (83)	22/30 (73)	.53

Abbreviations: CFIR, Consolidated Framework for Implementation Research; MSK, Memorial Sloane Kettering; NA, not applicable; RT, radiotherapy.

^a^ Please note that the total number of responses may differ by question because not all survey respondents answered every question asked.

^b^ The *P* values refer to results of 2-tailed Fisher exact test comparing preimplementation vs postimplementation responses, with significance set at *P* < .05.

With respect to characteristics of the intervention, there were no significant differences before or after implementation in the proportion of physicians who rated the strength and quality of evidence supporting use of 5 or fewer fractions for nonspine bone metastases as strong or moderately strong (24 of 29 physicians [83%] vs 26 of 30 physicians [87%]; *P* = .73, Fisher exact test). Although the proportion of physicians who rated the effectiveness of communication within the academic-community partnership as effective or mostly effective increased (12 of 29 physicians [41%] vs 19 of 30 physicians [63%]), this change was not significant (*P* = .12, Fisher exact test). After implementation, 26 of 29 physicians (90%) agreed or strongly agreed that use of short-course RT for nonspine bone metastases appeared to have more advantages than disadvantages.

For the inner setting, 28 of 29 physicians (97%) reported that staff members in their organization were receptive or very receptive to change preimplementation, and 29 of 29 physicians (100%) reported that staff members were receptive to change postimplementation. Postimplementation, 22 of 29 physicians (76%) agreed or completely agreed that senior leadership sought to increase patient participation and improve patient education regarding short-course RT (ie, leadership engagement). For the outer setting, postimplementation, 28 of 29 physicians (97%) agreed or strongly agreed that using 5 or fewer fractions to treat nonspine bone metastases takes patient needs and preferences into consideration. Finally, regarding the characteristics of the individuals, there was a modest but nonsignificant decrease in the percentage of physicians who reported feeling very confident in treating nonspine bone metastases preimplementation and postimplementation (24 of 29 physicians [83%] vs 22 of 30 physicians [73%]; *P* = .53, Fisher exact test).

#### Implementation Outcomes by ALIGNMENT Strategy

Postimplementation, 30 physicians rated the usefulness of ALIGNMENT strategies. Overall, guideline dissemination was the most highly rated strategy in terms of usefulness, with 26 physicians (87%) stating that they agreed that the strategy was useful in practice, followed by audit-and-feedback (18 physicians [60%]), and then eConsults (17 physicians [57%]).

### Guideline Distribution

In the postimplementation survey, most physicians (29 physicians [97%]) reported having either skimmed or carefully read the multidisciplinary consensus guidelines. Of the 3 strategies, guideline dissemination had the most favorable implementation outcomes (Table 4). By the end of the study, 23 physicians (77%) reported being likely to recommend the guidelines to other oncologists.

**Table 4.** Implementation Outcomes by ALIGNMENT Strategy

Outcome	Guidelines			eConsults			Audit and feedback		
	Physicians, No. (%)^a^		*P* value^b^	Physicians, No. (%)^a^		*P* value^b^	Physicians, No. (%)^a^		*P* value^b^
	Presurvey	Postsurvey		Presurvey	Postsurvey		Presurvey	Postsurvey	
Acceptability	22 (76)	28 (93)	.08	20 (69)	23 (77)	.57	21 (72)	27 (90)	.10
Appropriateness	23 (79)	27 (90)	.30	25 (86)	24 (80)	.73	19 (66)	27 (90)	.03
Feasibility									
Implementable	26 (90)	28 (93)	.67	24 (83)	26 (87)	.73	17 (59)	28 (93)	.002
Easy to use	24 (83)	27 (90)	.47	25 (86)	24 (80)	.73	13 (45)	28 (93)	<.001

^a^ Table values represent the number and percentage of physicians who agreed or completely agreed with each question. Please note that the preimplementation had 29 total respondents, whereas the postimplementation survey had 30 total respondents.

^b^ The *P* values refer to results of 2-tailed Fisher exact test with significance set at *P* < .05.

### Audit and Feedback

Personalized audit-and-feedback reports were also highly acceptable, appropriate, and feasible, with significant increases preimplementation vs postimplementation in physician perceptions of appropriateness (19 of 29 physicians [66%] vs 27 of 30 physicians [90%]; *P* = .03, Fisher exact test) and feasibility (implementable, 17 of 29 physicians [59%] vs 28 of 30 physicians [93%]; *P* = .002, Fisher exact test; easy to use, 13 of 29 physicians [45%] vs 28 of 30 physicians [93%]; *P* < .001, Fisher exact test). When asked about the optimal frequency for audit-and-feedback reports, 20 physicians (67%) preferred quarterly reports.

### eConsults

Overall, only 4 eConsults were requested by physicians during the study period. Expert time spent ranged between 7.5 to 15 minutes, while response time back to the requesting physician ranged between 14 to 79 minutes. Although 13 physicians (43%) reported having at least 1 difficult case or question related to the treatment of bone metastases, only 10 (33%) preferred eConsults, whereas 15 (50%) preferred consulting a friend or colleague. Of the 13 survey respondents who reported having a difficult case or clinical question about bone metastases in the prior 3 months, all reported having reached out to another physician within their institution or using a web-based reference or guideline. In addition, although 23 physicians (76%) responded that they reach out to their institutional colleagues often or almost always, 16 (53%) reported having never reached out to physicians at MSK, despite 20 physicians (67%) reporting before implementation that they prefer an email-based eConsults system over phone-based communication for discussing difficult cases.

Despite low utilization rates, eConsults had high levels of acceptability, appropriateness, and feasibility overall (Table 4). Among those who did use eConsults, all physicians gave the highest rating (10 out of 10) in terms of likelihood to recommend.

## Discussion

To our knowledge, this stepped-wedge, cluster randomized quality improvement study conducted across 3 clinical sites within an existing academic-community partnership represents the first study to prospectively assess the effectiveness of implementation strategies to improve the adoption of evidence-based short-course RT. A multicomponent set of implementation strategies had high levels of perceived acceptability, feasibility, and appropriateness among participating physicians, although use varied and effectiveness appeared somewhat limited. However, there was improvement overall in the desired practice associated with calendar time.

Collectively, these findings demonstrate the continued need to identify and examine robust strategies for promoting practice change. Of the 3 strategies studied, dissemination of guidelines was perceived by physicians to be the most useful strategy in practice. Perhaps this reflects an intervention-level barrier related to complexity, particularly regarding the use of more complex treatments that also can lead to shorter courses of radiation. These findings are consistent with prior literature suggesting that clinical guidelines can be perceived as successful when introduced in the appropriate context and with the appropriate method for development and dissemination^19^; however, more active strategies may be needed to promote practice change.

eConsults were designed to provide tailored case-based recommendations regarding radiation treatment in a more accessible and timely manner than tumor boards.^36^ Before implementation, the eConsult system was the second most highly ranked strategy in terms of usefulness, and among physicians submitting eConsults, usability was very high. However, eConsults were infrequently underutilized in our cohort, despite 43% of physicians reporting at least 1 difficult case or clinical question related to the treatment of nonspine bone metastases during the study period. Physicians more often preferred reaching out to a friend or colleague within their own institution. These findings suggest that physicians’ stated preferences may not adequately reflect success in clinical practice. We hypothesize that challenges to eConsult use by community-based physicians include physician time, inertia, and institutional culture and that opportunities to enhance eConsult use may include academic detailing of internal domain-specific champions (when available), strengthening physician networks and reducing cross-institutional barriers to communication, and finding ways to incentivize this effort among busy practicing physicians. Nonetheless, future studies are needed to further assess barriers and determine whether strategies mitigating them improve use.

Audit and feedback represents a more active strategy that has been found effective in promoting practice change in other medical specialties.^37^ One study^38^ ranked audit and feedback as second of 73 strategies in terms of importance and eighth in terms of scalability. In addition, several economic evaluations suggest that audit-and-feedback–based interventions have high potential to be cost-effective.^39^ However, multiple recent studies investigating audit and feedback specifically for deimplementation of low-value care have shown less to no effect.^40,41^ In the present study, although initial perceptions regarding its usefulness were relatively low compared with guidelines and eConsults, physicians’ perceptions significantly improved postimplementation, suggesting promise. Physicians did report preferring reports quarterly, perhaps to monitor results of one’s practice improvement efforts, whereas in the current study they were provided only once. This suggests lack of effectiveness could relate to inadequate dose of the strategy, consistent with other studies.^42^

Our study offers important insights into leveraging an academic-community partnership to help improve the guideline-concordant delivery of subspecialty care. There is much enthusiasm about the potential for such partnerships to improve research and practice,^34,43^ and the past decade has seen an increase in hospital mergers and affiliations resulting in expanding health networks.^44^ However, evidence to date suggests limited improvements in quality of care.^45^ In this study, we successfully developed and tested a multicomponent set of strategies that informed physicians of an area for practice improvement while providing resources for practice change; this model resulted in high perceptual implementation outcomes. Although efforts are needed to enhance effectiveness, either through modifying existing strategies or incorporating novel ones, we expect that the ALIGNMENT study approach and experience will inform initiatives within other academic-community partnerships to help improve and standardize complex oncologic care.

### Limitations

These findings should be interpreted in the context of the study’s limitations. First, participating sites were part of a single academic-community partnership, which may limit generalizability. Second, we relied on physician self-report of review of audit and feedback and the consensus guidelines (rather than electronic read receipt confirmation). Third, physician identifiers were not included in surveys, which limits preimplementation and postimplementation paired testing and linking perceptual outcomes with practice outcomes. Fourth, possible explanations for observed practice improvement over time include (1) a potential Hawthorne effect, whereby physicians may have changed their practice because of their awareness of the study (which was grant funded and began with a consensus process involving the site champions, as described elsewhere)^24^ rather than the study implementation strategies being tested, which is a challenge not uncommon in quality improvement given the desire to engage stakeholders (including site champions in the current study); (2) contamination between clinical sites, which was also possible in a sequential rollout design such as the stepped wedge (although no physicians changed to another institution during the study period, facilitating high adherence to study design); (3) an unrelated secular trend related to anticipation of a bundled payment model; and (4) an unrelated secular trend related to the emergence of randomized data supporting complex RT, although complex RT itself did not increase (data not shown).^11,46^ Nonetheless, our results emphasize the importance of formally evaluating quality improvement initiatives.^47^

## Conclusions

In this prospective, stepped-wedge, cluster randomized, quality improvement study conducted across an existing academic-community partnership, a multicomponent set of 3 implementation strategies was not directly associated with increased use of short-course RT. However, practice did improve overall with time, perhaps owing to secular trends or physician awareness of the study. These findings demonstrate the continued need to examine the optimal approaches for encouraging practice change and improving care quality for patients with nonspine bone metastases.

## Notes

Supplement 1. **eAppendix 1.** Supplemental Methods
eReferences
**eAppendix 2.** Pre- and Post-Implementation Survey Questions and Results **eTable 1.** Physician Characteristics **eTable 2.** Cluster Summaries—Patient Characteristics **eTable 3.** Cluster Summaries—Lesion-Level Characteristics

Supplement 2.
Data Sharing Statement
