# Supplementary material for: Implementation Strategies to Promote Short-Course Radiation for Bone Metastases
Source: JAMA Netw Open. 2024 May 24;7(5):e2411717. doi: 10.1001/jamanetworkopen.2024.11717 (PMC11127116; doi:10.1001/jamanetworkopen.2024.11717)
Supplement: Supplement 1. — eAppendix 1. Supplemental Methods eReferences eAppendix 2. Pre- and Post-Implementation Survey Questions and Results eTable 1. Physician Characteristics eTable 2. Cluster Summaries—Patient Characteristics eTable 3. Cluster Summaries—Lesion-Level Characteristics [file jamanetwopen-e2411717-s001.pdf]

Supplemental Online Content

Gillespie EF, Santos PMG, Curry M, et al. Implementation strategies to promote short-course radiation for bone metastases. *JAMA Netw Open*. 2024;7(5):e241171. doi:10.1001/jamanetworkopen.2024.11717

eAppendix 1. Supplemental Methods
eReferences
eAppendix 2. Pre- and Post-Implementation Survey Questions and Results
eTable 1. Physician Characteristics
eTable 2. Cluster Summaries—Patient Characteristics
eTable 3. Cluster Summaries—Lesion-Level Characteristics

This supplemental material has been provided by the authors to give readers additional information about their work.

© 2024 Gillespie EF et al. *JAMA Network Open.*

eAppendix 1. Supplemental Methods

Implementation Strategies

Guidelines (Distribution of Educational Materials)

The first component of the intervention was the distribution of consensus recommendations developed by a multidisciplinary team of physicians from MSK and each Alliance site, as previously described.[1] This strategy is supported by data suggesting that locally-derived guidelines generally have greater impact than national ones, and that mode of delivery matters.[2] Additionally, oncologists seek information from experts even in the absence of definitive evidence from multiple randomized trials.[3] Of note, guidelines were delivered *actively*, including oral presentations (via zoom by an MSK metastatic radiation clinical expert and answering eConsults) to both the radiation oncology and medical oncology physician teams during each institution's assigned rollout, then followed by individual emails with guideline PDF attached.

eConsults

The second component of the intervention was practice facilitation—an implementation strategy that has been identified as one of the top innovator ideas to promote uptake of evidence-based practice.[4] In this study, practice facilitation was implemented via the e-Consults platform: a secure e-mail-based system whereby MSK Cancer Alliance physicians could ask clinical questions and send case information (including screenshots of imaging) for expert feedback. The inbox was staffed by MSK metastatic disease specialists who were compensated for assigned daily coverage ($25/day) and individual consult case responses ($25/response). All responses occurred within 24 hours and included either recommendations for treatment or a suggestion to present the case at the weekly MSK Bone Metastases tumor board.

Audit-and-Feedback

The third component of the intervention consisted of audit-and-feedback reports for each physician with peer comparisons. As an implementation strategy with both high efficacy and scalability, audit-and-feedback has an effect size of audit-and-feedback of approximately 5 percent.[4,5] In our study, each personalized audit-and-feedback report included graphic and numerical data about an individual physician's adherence to consensus recommendations (i.e., use of ≤5 fractions), as well as personalized

© 2024 Gillespie EF et al. *JAMA Network Open.*

50% improvement targets. The proportion of treatments in concordance with the consensus recommendation for an individual physician were presented anonymously alongside peers within their institution as well as the site average for MSK. The reports were distributed via email from physician leadership within their department at the beginning of the intervention period. Performance was based on treatment data from January to October 2020.

Physician Surveys

Assessment of Context and Determinants

An online survey tool informed by the Consolidated Framework for Implementation Science Research (CFIR)[6] was developed to define contextual determinants and further ensure generalizability of findings. Characteristics of the **intervention** (i.e., consensus recommendation of ≤5 fractions) were assessed by querying evidence strength and relative advantage in the physician survey. Characteristics of **individuals** (i.e., participating physicians) were assessed based on level of specialization within radiation oncology, patient volume, and confidence treating bone metastases. Years of experience was also collected based on medical school graduation year. The **inner setting** was assessed using the "staff receptiveness to change" item from Organizational Readiness for Change Assessment mapped from CFIR.[6,7] Lastly, the **outer setting** was assessed at the practice-level by measuring the "effectiveness of communication" between clinical sites using the Agency for Healthcare Research and Quality Clinical Community Relationship measure.[8]

© 2024 Gillespie EF et al. *JAMA Network Open.*

eREFERENCES

1. Gillespie EF, Mathis NJ, Vaynrub M, et al. Multidisciplinary Treatment of Non-Spine Bone Metastases: Results of a Modified Delphi Consensus Process. *Clin Transl Radiat Oncol*. 2022;35:76-83. doi:10.1016/j.ctro.2022.04.009

2. Grimshaw JM, Russell IT. Effect of clinical guidelines on medical practice: a systematic review of rigorous evaluations. *The Lancet*. 1993;342(8883):1317-1322.

3. Dillmon M, Goldberg JM, Ramalingam SS, Mayer RJ, Loehrer P, Van Poznak C. Clinical practice guidelines for cancer care: utilization and expectations of the practicing oncologist. *Journal of Oncology Practice*. 2012;8(6):350-353.

4. Stewart RE, Williams N, Byeon YV, et al. The clinician crowdsourcing challenge: using participatory design to seed implementation strategies. *Implementation Science*. 2019;14(1):1-8.

5. Jamtvedt G, Young JM, Kristoffersen DT, O'Brien MA, Oxman AD. Does telling people what they have been doing change what they do? A systematic review of the effects of audit and feedback. *BMJ Quality & Safety*. 2006;15(6):433-436.

6. Damschroder LJ, Reardon CM, Widerquist MAO, Lowery J. The updated Consolidated Framework for Implementation Research based on user feedback. *Implementation Science*. 2022;17(1):1-16.

7. Helfrich CD, Li YF, Sharp ND, Sales AE. Organizational readiness to change assessment (ORCA): development of an instrument based on the Promoting Action on Research in Health Services (PARIHS) framework. *Implementation science*. 2009;4(1):1-13.

8. Introduction. Accessed April 17, 2023. https://www.ahrq.gov/prevention/resources/chronic-care/clinical-community-relationships-measures-atlas/ccrm-atlasintro.html

© 2024 Gillespie EF et al. *JAMA Network Open.*

eAppendix 2. Pre- and Post-Implementation Survey Questions and Results

A) Pre-Implementation

1. How confident do you feel in your practice treating bone metastases?

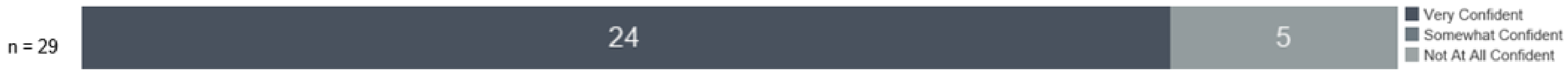

2. Please rate the effectiveness of communication between physicians in your organization and physicians at MSK

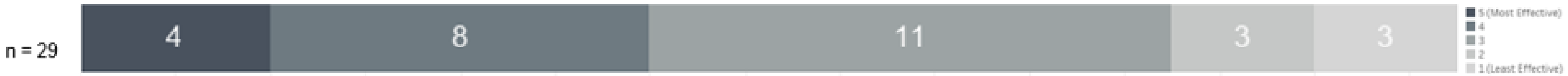

3. Please rank the strategies below in order of how useful you think they would be for your clinical practice.

Multidisciplinary consensus recommendations

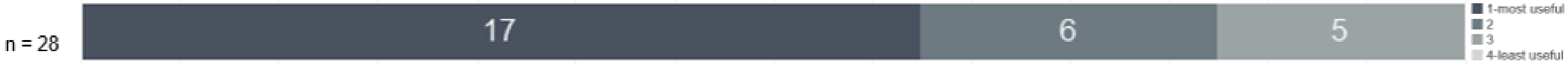

Access to weekly MSK tumor boards

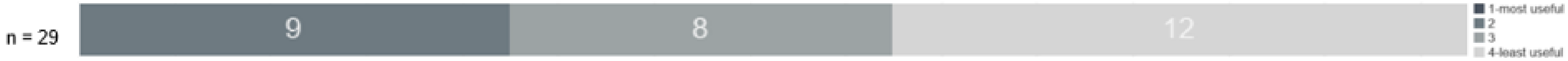

Personalized feedback report on practice patterns

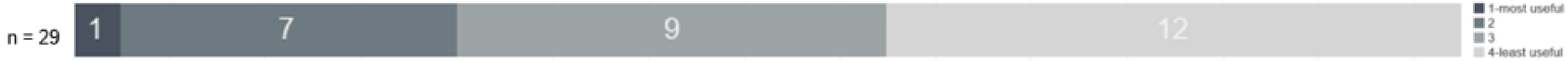

Access to an email consultation (e-Consults) system

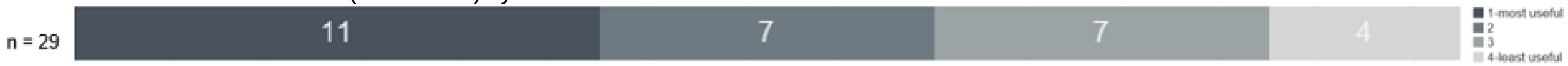

4. Please tell us how much you agree or disagree with the following statements about the recommendation to use 5 or fewer (≤5) RT fractions for non-spine bone metastases

I welcome the recommendation

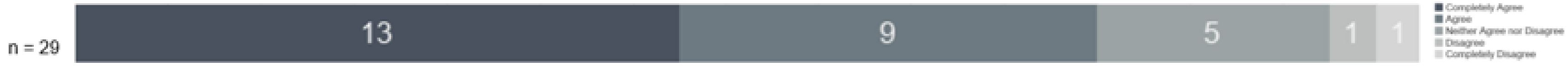

© 2024 Gillespie EF et al. *JAMA Network Open.*

The recommendation seems like a good match

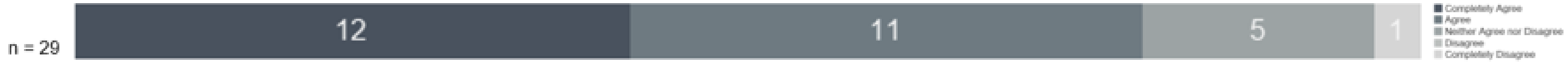

The recommendation seems implementable

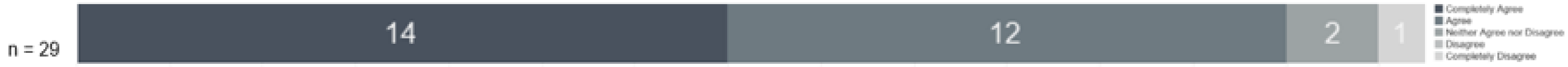

The recommendation seems easy to use

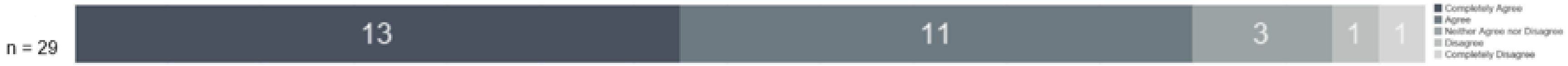

5. Please tell us how much you agree or disagree with the following statements about receiving personalized feedback on your clinical practice compared to your peers.

I welcome receiving personalized feedback on my practice

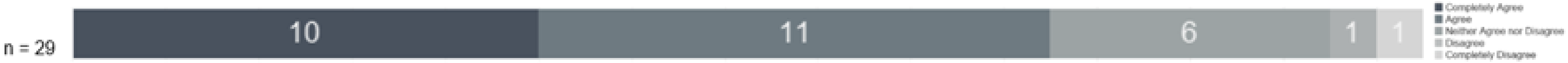

Receiving personalized feedback seems like a good match

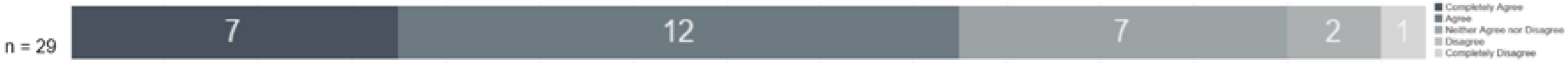

Receiving personalized feedback seems implementable

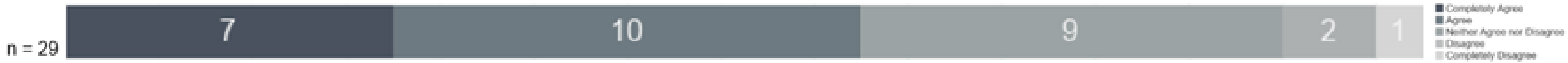

Receiving personalized feedback seems easy to use

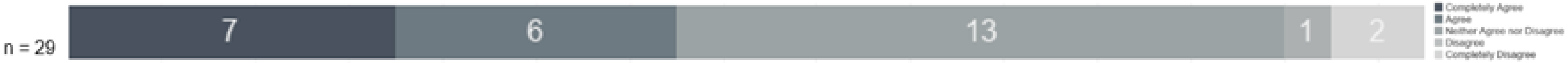

6. Please tell us how much you agree or disagree with the following statements about e-Consults, which is a platform for email communications with MSK physicians about clinical questions.

I welcome having access to e-Consults

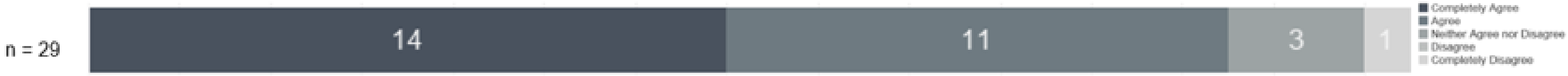

Access to e-Consults seems like a good match

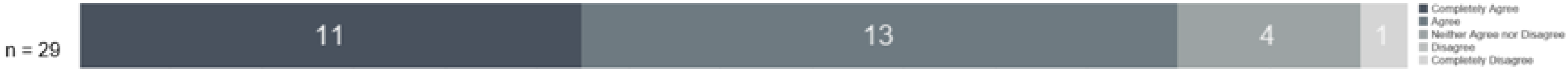

© 2024 Gillespie EF et al. *JAMA Network Open.*

Access to e-Consults seems implementable

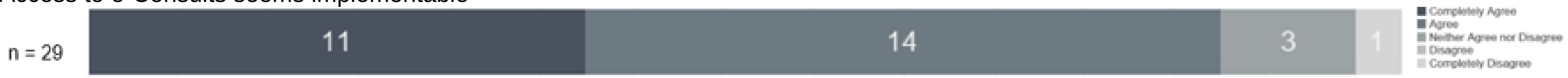

Access to e-Consults seems easy to use

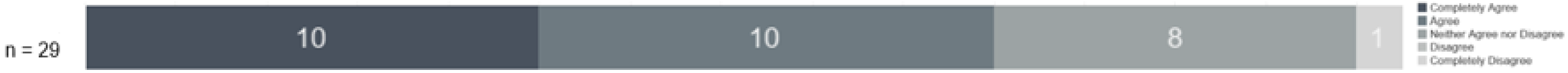

7. How would you rate the need for personalized feedback and access to e-Consults within your organization?

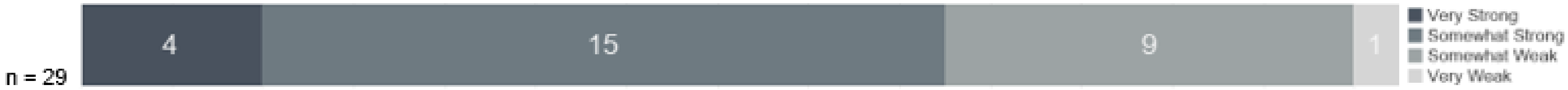

8. In general, how receptive are staff members in your organization to change in clinical processes?

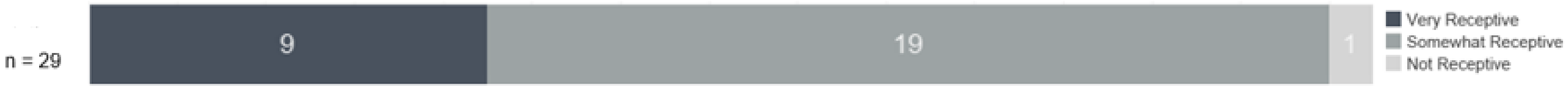

© 2024 Gillespie EF et al. *JAMA Network Open.*

eAppendix 2. Pre- and Post-Implementation Survey Questions and Results

B) Post-Implementation

1. How confident do you feel in your practice treating bone metastases?

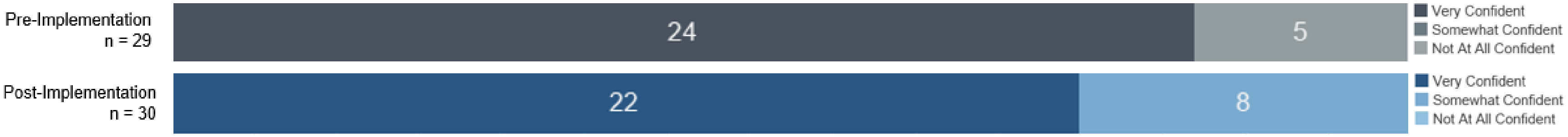

2. Based on your assessment of the evidence, please rate the strength of the evidence for using five or fewer 4 (≤5) fractions of RT to treat non-spine bone 3 metastases on a scale of 1 to 5 (1- weak evidence, 5- strong evidence)

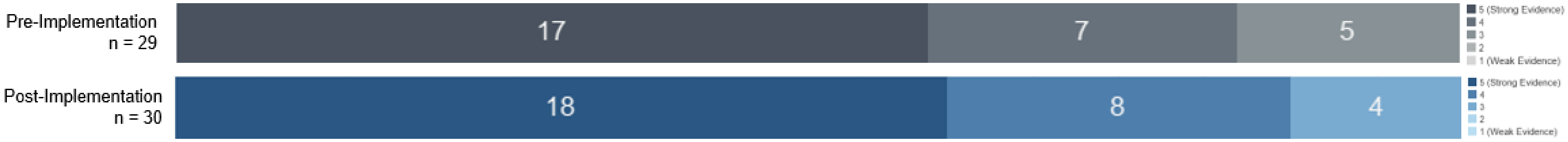

3. Please rate the effectiveness of communication between physicians in your organization and physicians at MSK:

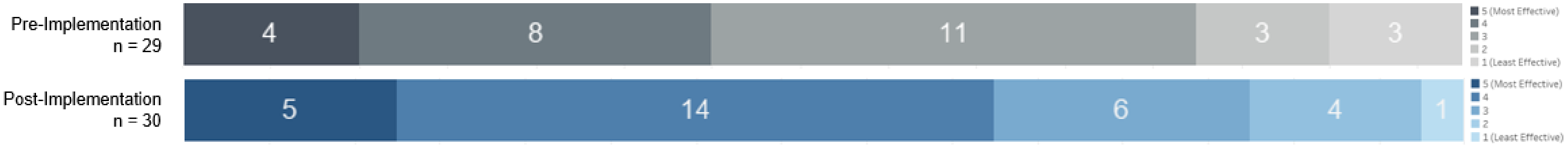

4. Using five or fewer (≤5) fractions of RT to treat non-spine bone metastases appears to have more advantages than disadvantages.

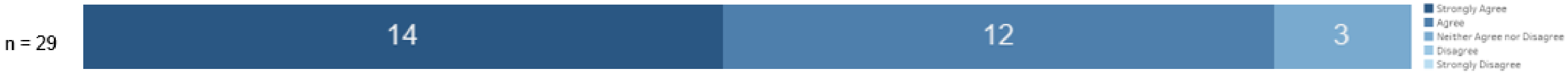

© 2024 Gillespie EF et al. *JAMA Network Open.*

5. **Using five or fewer (≤5) fractions of RT to treat non-spine bone metastases takes into consideration the needs and preferences of patients.**

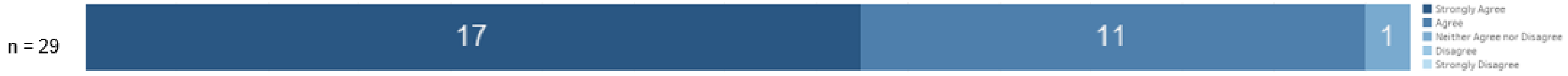

6. **Please tell us how much you agree or disagree with the following statements about the resources provided to you as part of the MSK Cancer Alliance ALIGNMENT intervention:**

The multidisciplinary recommendations were useful

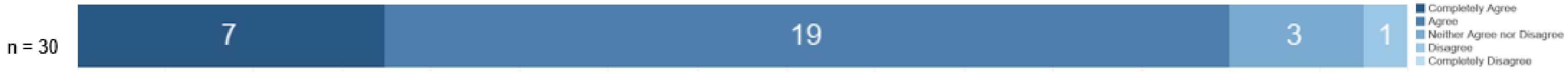

Having access to weekly MSK tumor boards was useful

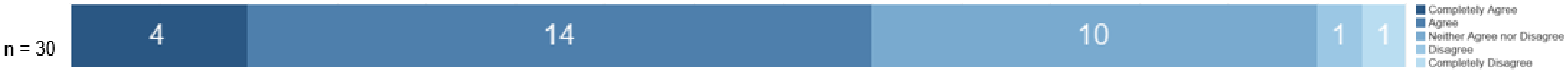

Receiving a personalized feedback report on practice patterns was useful

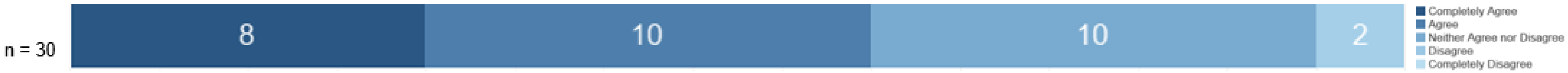

Having access to an e-mail consulting (e-consults) system was useful

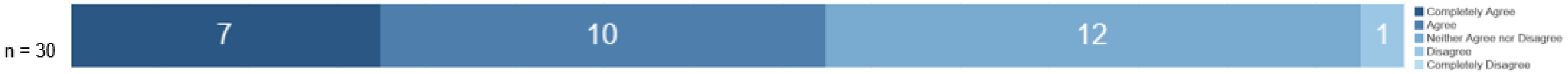

7. **Please rank the strategies below in order of how useful you think they would be for your clinical practice (Pre-Implementation)**

Multidisciplinary recommendations

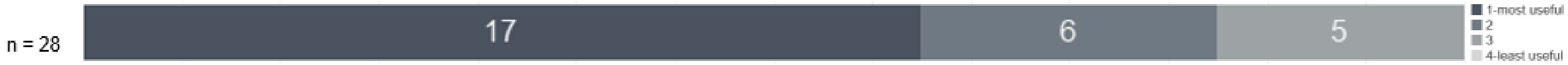

Access to weekly MSK tumor boards

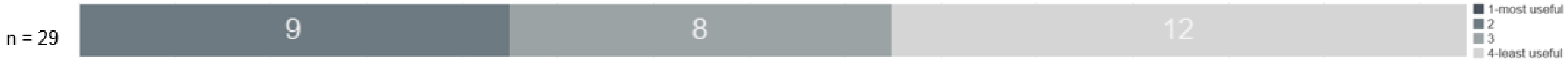

Personalized feedback report on practice patterns

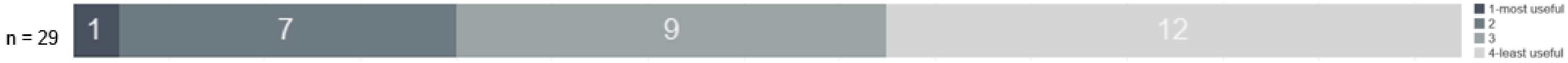

© 2024 Gillespie EF et al. *JAMA Network Open.*

Access to an e-mail consultation (e-Consults) system

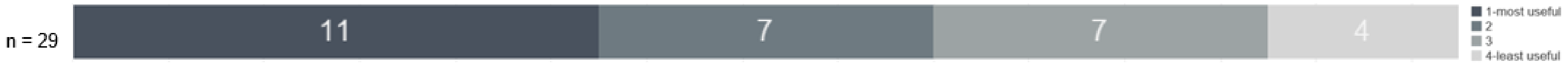

8. Did you review the provided consensus recommendations?

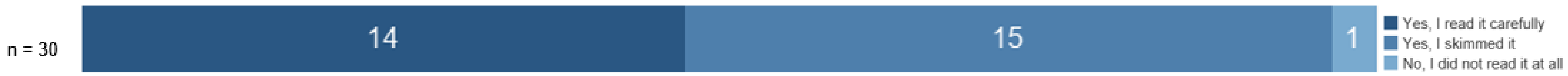

9. How likely are you to recommend the consensus recommendations to other radiation oncologists?

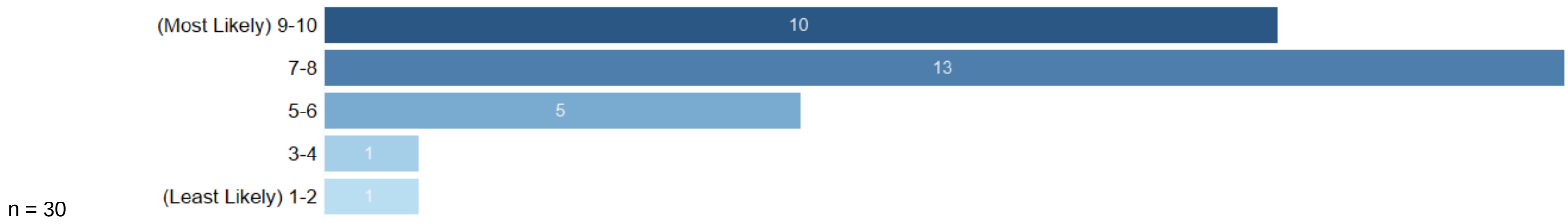

10. One of the consensus recommendations was to use five or fewer (≤5) fractions of RT for non-spine bone metastases. Please tell us how much currently agree or disagree with following

I welcome the recommendation

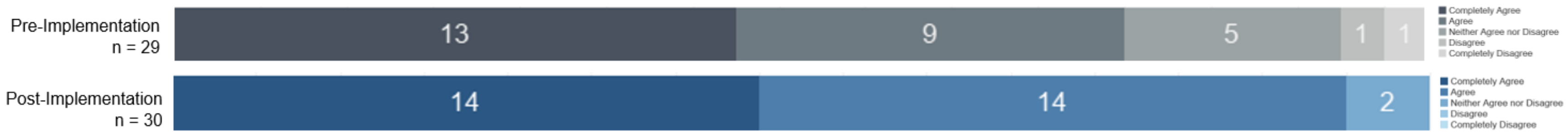

The recommendation seems like a good match

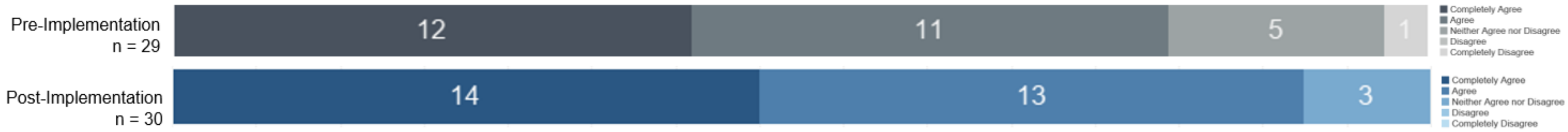

© 2024 Gillespie EF et al. *JAMA Network Open.*

The recommendation seems implementable

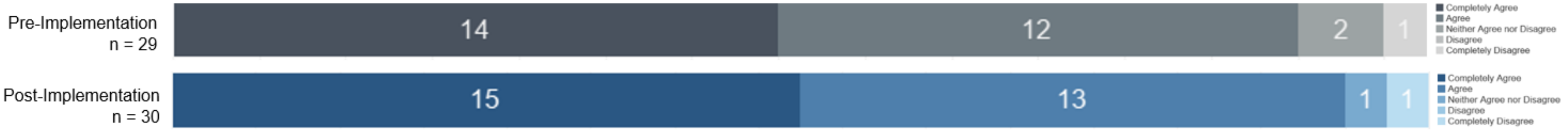

The recommendation seems easy to use

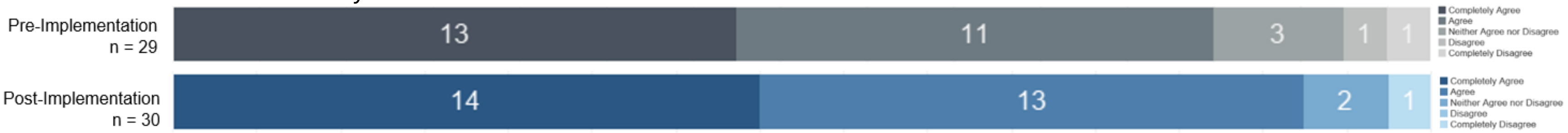

11. Three months ago, you received a report with personalized feedback on your clinical practice regarding your use of five or fewer (≤5) fractions of RT. The next section is about this resource. Please tell us how much you currently agree or disagree with the following statements about this resource

I welcome receiving personalized feedback on my practice

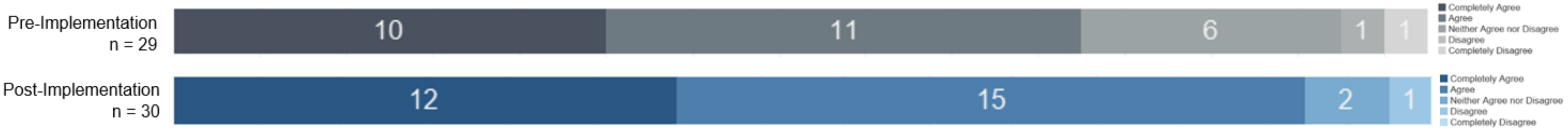

Receiving personalized feedback seems like a good match

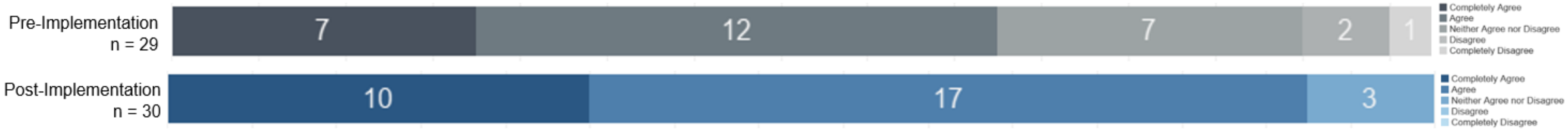

Receiving personalized feedback seems implementable

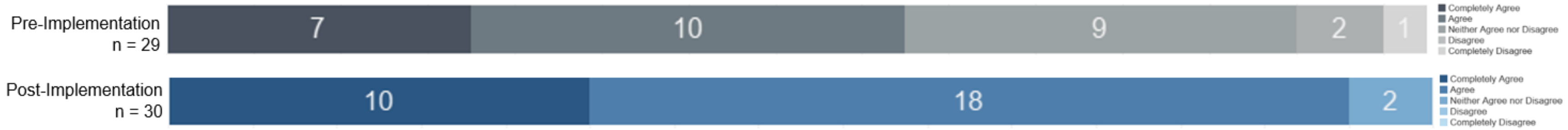

Receiving personalized feedback seems easy to use

© 2024 Gillespie EF et al. *JAMA Network Open.*

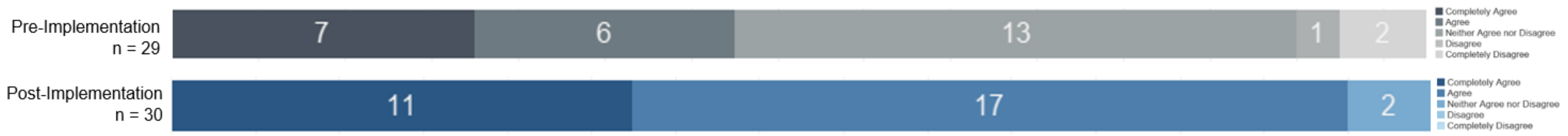

12. What would be the optimal frequency for practice feedback reports?

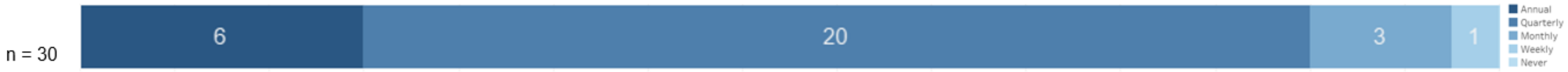

13. In the past 3 months, approximately how many patients with bone metastases did you treat?

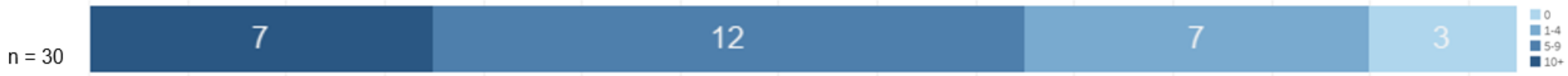

14. In the past 3 months, have you had any difficult cases or clinical questions related to the treatment of bone metastases?

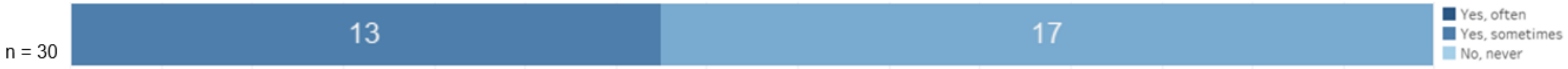

15. In the past 3 months, if you had clinical questions about bone metastases, how often did you reach out to a:

Physician at MSK

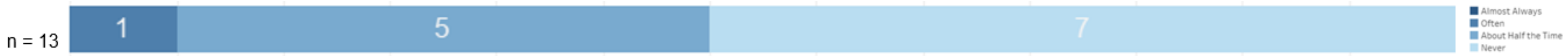

Physician at another institution besides MSK

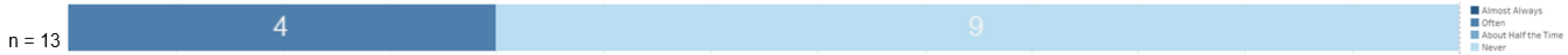

Physician within my institution

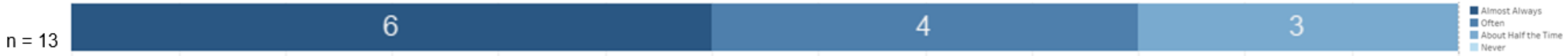

Web-based reference or guideline

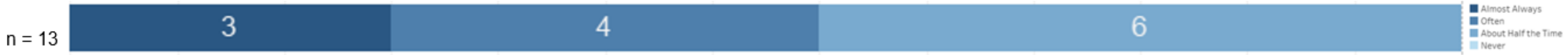

© 2024 Gillespie EF et al. *JAMA Network Open.*

16. Please tell us how much you currently agree or disagree with the following statements about e-Consults:

Access to e-Consults seems easy to use

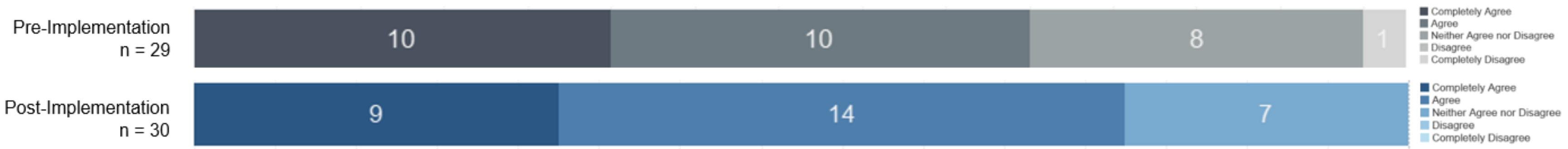

Access to e-Consults seems implementable

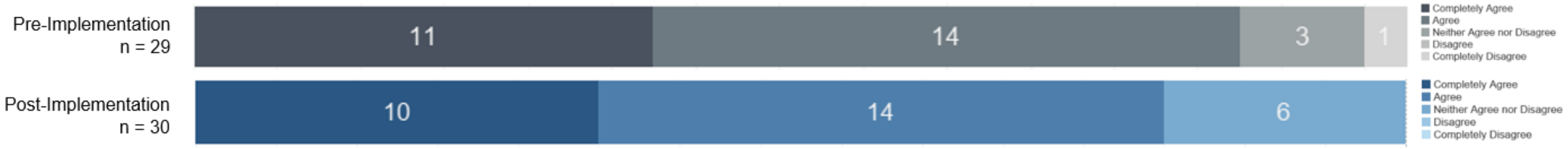

Access to e-Consults seems like a good match

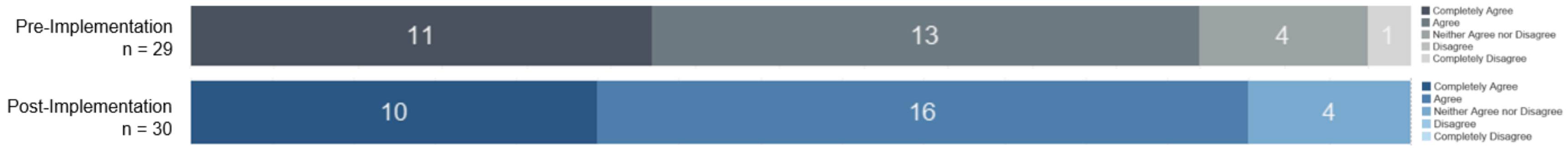

I welcome having access to e-Consults

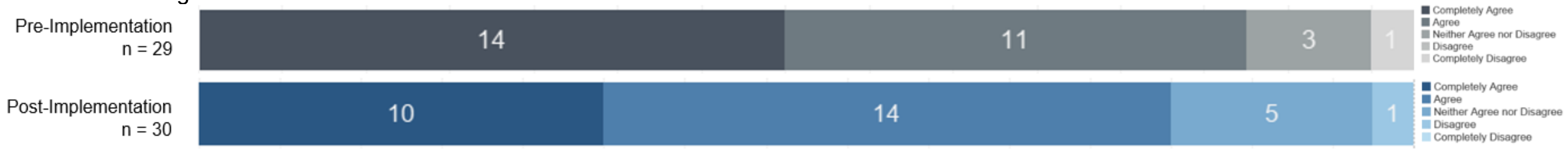

17. Compared with phone-based communication (phone call or text message) with a physician colleague at MSK, how would you rate email-based communication:

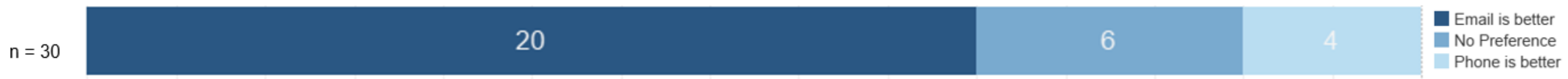

© 2024 Gillespie EF et al. *JAMA Network Open.*

18. **For questions about difficult clinical cases, would you prefer reaching out to an MSK specialist through e-Consults or to a friend/colleague?**

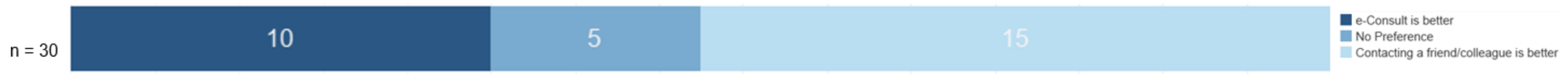

19. **Senior leadership in your organization seeks ways to improve patient education and increase patient participation in treatment.**

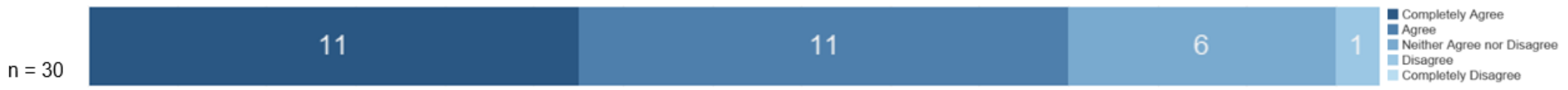

20. **In general, how receptive are staff members in your organization to change in clinical processes?**

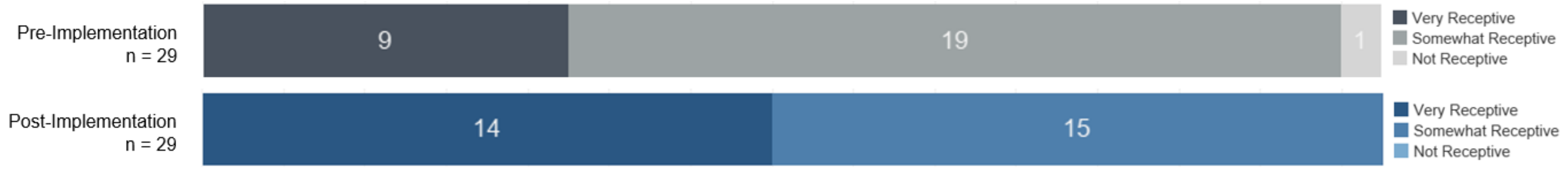

21. **How many disease sites do you usually treat?**

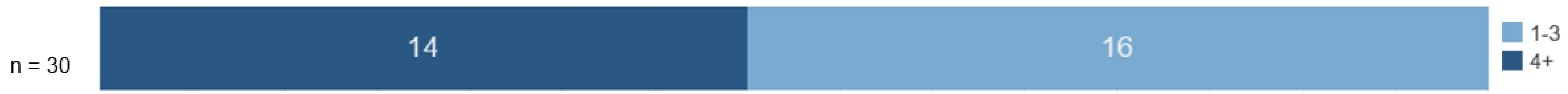

© 2024 Gillespie EF et al. *JAMA Network Open.*

eTable 1. Physician Characteristics

Characteristics	Overall
No. (%) of physicians	N=45
Institution, n (%)	
Hartford Healthcare	21 (46.7%)
Lehigh Valley Health Network	10 (22.2%)
Miami Cancer Institute	14 (31.1%)
Number of patients, median (IQR)	13 (7, 20)
Complied at baseline with recommendation, n (%)	
Yes	8 (18%)
Years of experience in 2021, median (IQR)	21 (10, 35)

© 2024 Gillespie EF et al. *JAMA Network Open.*

eTable 2. **Cluster Summaries—Patient Characteristics**

	HHC			LVHN			MCI		
**Characteristic**	**Overall**, N = 396	**Pre**, N = 266	**Post**, N = 130	**Overall**, N = 182	**Pre**, N = 139	**Post**, N = 43	**Overall**, N = 260	**Pre**, N = 223	**Post**, N = 37
**Age at Treatment Start**[1]	67 (60, 75)	67 (60, 75)	67 (60, 76)	67 (61, 76)	67 (60, 76)	68 (62, 77)	66 (54, 73)	66 (55, 73)	64 (53, 75)
Unknown	4	0	4	0	0	0	0	0	0
**Sex**									
F	175 (44%)	115 (43%)	60 (47%)	88 (48%)	64 (46%)	24 (56%)	140 (54%)	120 (54%)	20 (54%)
M	219 (56%)	151 (57%)	68 (53%)	94 (52%)	75 (54%)	19 (44%)	120 (46%)	103 (46%)	17 (46%)
Unknown	2	0	2	0	0	0	0	0	0
**Race**									
Asian	6 (1.5%)	5 (1.9%)	1 (0.8%)	3 (1.7%)	3 (2.2%)	0 (0%)	0	0	0
Black	31 (8.0%)	23 (8.7%)	8 (6.4%)	13 (7.2%)	11 (8.0%)	2 (4.7%)	27 (10%)	18 (8.1%)	9 (24%)
Other	30 (7.7%)	18 (6.8%)	12 (9.6%)	8 (4.4%)	6 (4.4%)	2 (4.7%)	27 (10%)	25 (11%)	2 (5.4%)
White	321 (83%)	217 (83%)	104 (83%)	156 (87%)	117 (85%)	39 (91%)	206 (79%)	180 (81%)	26 (70%)
Unknown	8	3	5	2	2	0	0	0	0
**Patient Ethnicity (Hispanic)**									
N	354 (92%)	246 (93%)	108 (90%)	167 (92%)	128 (93%)	39 (91%)	93 (36%)	79 (35%)	14 (38%)
Y	30 (7.8%)	18 (6.8%)	12 (10%)	14 (7.7%)	10 (7.2%)	4 (9.3%)	167 (64%)	144 (65%)	23 (62%)
Unknown	12	2	10	1	1	0	0	0	0
**ECOG**									
0	123 (32%)	82 (32%)	41 (33%)	45 (25%)	40 (29%)	5 (12%)	46 (18%)	32 (14%)	14 (38%)
1	173 (45%)	118 (46%)	55 (44%)	58 (33%)	41 (30%)	17 (41%)	111 (43%)	99 (44%)	12 (32%)
2	76 (20%)	50 (19%)	26 (21%)	57 (32%)	42 (31%)	15 (37%)	68 (26%)	65 (29%)	3 (8.1%)
3	8 (2.1%)	6 (2.3%)	2 (1.6%)	12 (6.7%)	10 (7.3%)	2 (4.9%)	28 (11%)	20 (9.0%)	8 (22%)
4	1 (0.3%)	1 (0.4%)	0 (0%)	6 (3.4%)	4 (2.9%)	2 (4.9%)	7 (2.7%)	7 (3.1%)	0 (0%)
Unknown	15	9	6	4	2	2	0	0	0
**>5 metastatic sites**									
N	66 (17%)	44 (17%)	22 (18%)	34 (19%)	30 (22%)	4 (9.3%)	30 (12%)	24 (11%)	6 (16%)
Y	325 (83%)	222 (83%)	103 (82%)	148 (81%)	109 (78%)	39 (91%)	230 (88%)	199 (89%)	31 (84%)
Unknown	5	0	5	0	0	0	0	0	0
**Symptomatic from bone metastasis**									
N	34 (8.7%)	25 (9.4%)	9 (7.2%)	4 (2.3%)	2 (1.5%)	2 (4.7%)	3 (1.2%)	3 (1.3%)	0 (0%)
Y	357 (91%)	241 (91%)	116 (93%)	171 (98%)	130 (98%)	41 (95%)	257 (99%)	220 (99%)	37 (100%)
Unknown	5	0	5	7	7	0	0	0	0
**Prior surgery to treated metastasis**									
N	349 (89%)	239 (90%)	110 (88%)	163 (90%)	123 (88%)	40 (93%)	247 (95%)	211 (95%)	36 (97%)
Y	42 (11%)	27 (10%)	15 (12%)	19 (10%)	16 (12%)	3 (7.0%)	13 (5.0%)	12 (5.4%)	1 (2.7%)
Unknown	5	0	5	0	0	0	0	0	0

© 2024 Gillespie EF et al. *JAMA Network Open.*

	HHC			LVHN			MCI		
**Characteristic**	**Overall**, N = 396	**Pre**, N = 266	**Post**, N = 130	**Overall**, N = 182	**Pre**, N = 139	**Post**, N = 43	**Overall**, N = 260	**Pre**, N = 223	**Post**, N = 37
**Radioresistant Histology**									
Yes	106 (27%)	74 (28%)	32 (26%)	53 (32%)	45 (36%)	8 (20%)	44 (17%)	40 (18%)	4 (11%)
No	283 (73%)	190 (72%)	93 (74%)	114 (68%)	81 (64%)	33 (80%)	216 (83%)	183 (82%)	33 (89%)
Unknown	7	2	5	15	13	2	0	0	0
**Institution**									
HHC	396 (100%)	266 (100%)	130 (100%)	0	0	0	0	0	0
LVHN	0	0	0	182 (100%)	139 (100%)	43 (100%)	0	0	0
MCI	0	0	0	0	0	0	260 (100%)	223 (100%)	37 (100%)

[1]Median (IQR); n (%)

© 2024 Gillespie EF et al. *JAMA Network Open.*

eTable 3. **Cluster Summaries—Lesion-Level Characteristics**

	HHC			LVHN			MCI		
**Characteristic**	**Overall**, N = 395	**Pre**, N = 265	**Post**, N = 130	**Overall**, N = 183	**Pre**, N = 140	**Post**, N = 43	**Overall**, N = 260	**Pre**, N = 223	**Post**, N = 37
**Total Planned RT Dose (Gy)**[1]	30 (20, 30)	30 (20, 30)	30 (20, 30)	30.0 (20.0, 30.0)	30.0 (20.0, 30.0)	25.0 (20.0, 30.0)	20 (20, 30)	20 (20, 30)	20 (20, 30)
Unknown	4	0	4	0	0	0	0	0	0
**RT Technique**									
Complex (IMRT, SBRT)	56 (14%)	36 (14%)	20 (16%)	28 (15%)	20 (14%)	8 (19%)	44 (17%)	38 (17%)	6 (16%)
Simple (2D, 3D)	335 (86%)	229 (86%)	106 (84%)	155 (85%)	120 (86%)	35 (81%)	216 (83%)	185 (83%)	31 (84%)
Unknown	4	0	4	0	0	0	0	0	0
**Completed planned RT course**									
N	18 (4.6%)	9 (3.4%)	9 (7.2%)	5 (2.7%)	3 (2.2%)	2 (4.7%)	9 (3.5%)	9 (4.0%)	0 (0%)
Y	373 (95%)	257 (97%)	116 (93%)	177 (97%)	136 (98%)	41 (95%)	251 (97%)	214 (96%)	37 (100%)
Unknown	5	0	5	0	0	0	0	0	0

[1]n (%); Median (IQR)

© 2024 Gillespie EF et al. *JAMA Network Open.*
