# Supplementary material for: Implementation Strategies to Promote Short-Course Radiation for Bone Metastases
Source: JAMA Netw Open. 2024 May 24;7(5):e2411717. doi: 10.1001/jamanetworkopen.2024.11717 (PMC11127116; doi:10.1001/jamanetworkopen.2024.11717)
Supplement: Supplement 2. — Data Sharing Statement [file jamanetwopen-e2411717-s002.pdf]

Data Sharing Statement

Gillespie. Implementation Strategies to Promote Short-Course Radiation for Bone Metastases. *JAMA Netw Open*. Published May 16, 2024. doi:10.1001/jamanetworkopen.2024.11717

Data
Data available: Yes
Data types: Deidentified participant data
How to access data: Data will be available upon request.
When available: With publication

Supporting Documents
Document types: None

Additional Information
Who can access the data: Data will be available upon request from corresponding author.
Types of analyses: For specified purpose.
Mechanisms of data availability: With funding for data transfer.
